# Supplementary figures and images for: In-depth quantitative proteomics uncovers specie-specific metabolic programs in Leishmania (Viannia) species
Source: PLoS Negl Trop Dis. 2020 Aug 17;14(8):e0008509. doi: 10.1371/journal.pntd.0008509 (PMC7451982; doi:10.1371/journal.pntd.0008509)

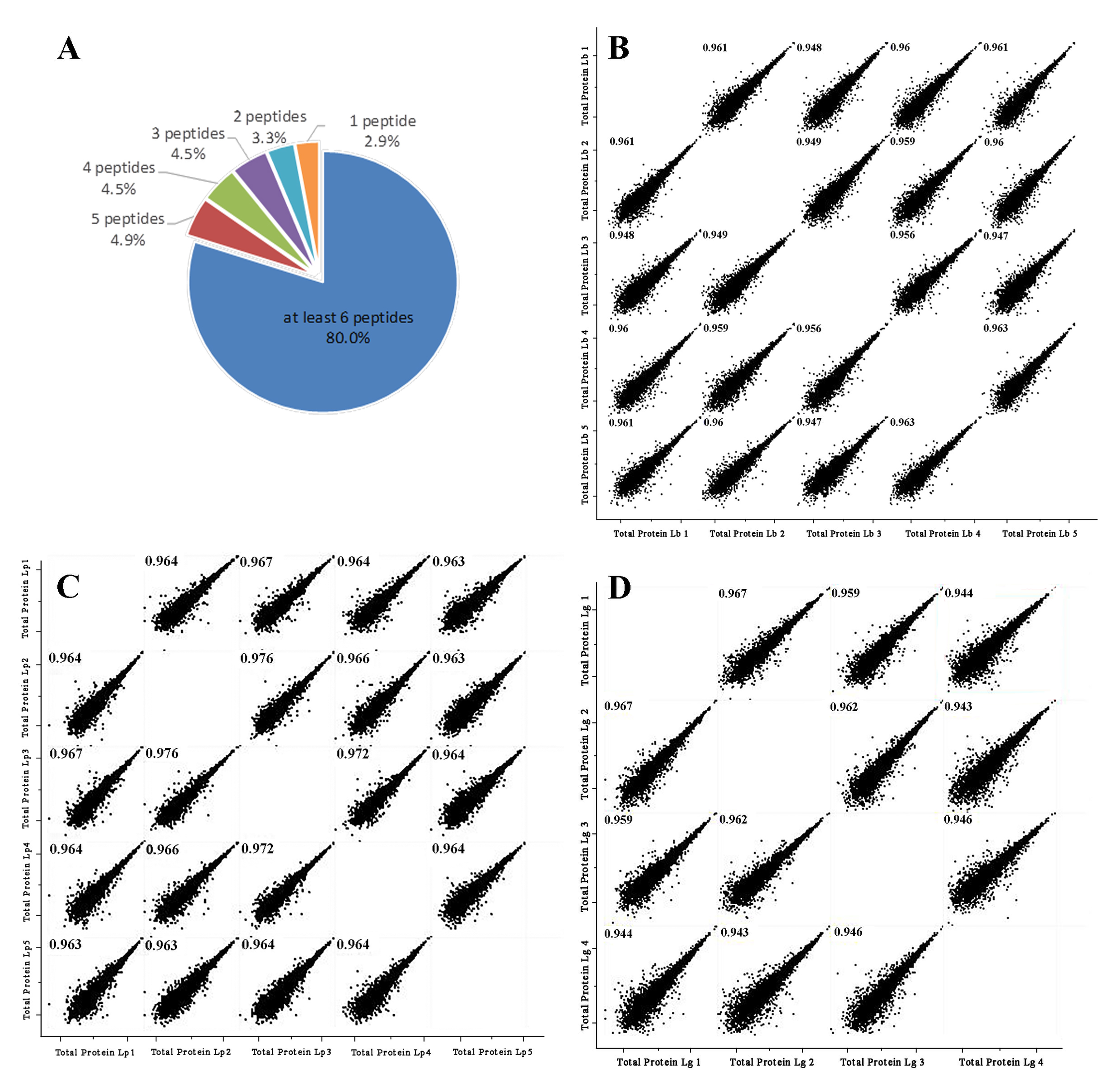

Supplement: S1 Fig — (A) 95% proteins were identified with at least three peptides. Profiles of Pearson’s correlation coefficient of protein abundance among (B) L. braziliensis replicates, (C) L. panamensis replicates, (D) L. guyanensis replicates. Plotted values represent the total protein fraction of each individual protein calculated by the total protein approach. (TIF) [file pntd.0008509.s001.tif]

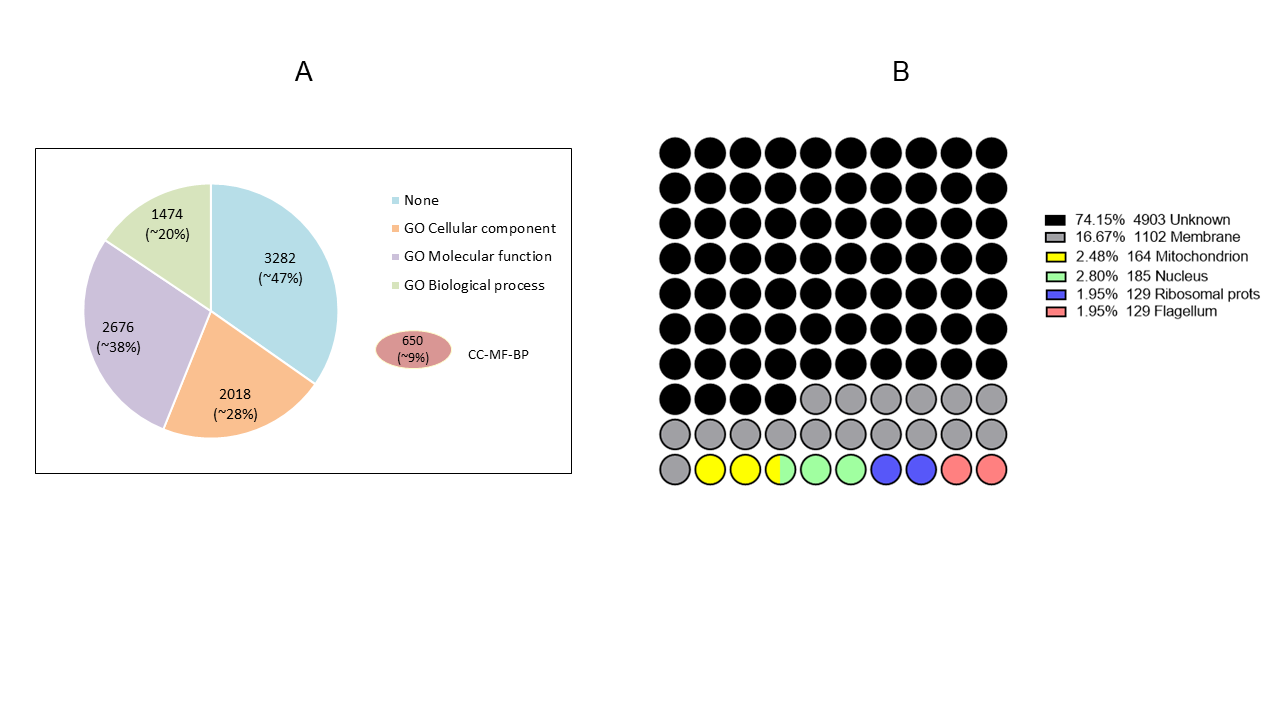

Supplement: S2 Fig — (A) Number and percentage of proteins in our dataset with any functional annotation for the main gene ontology (GO) categories of Cellular component, Molecular function and Biological process. (B) Number of proteins identified in each one of the main cellular components of Leishmania promastigotes, according to GO annotation for cellular component. (TIF) [file pntd.0008509.s002.tif]

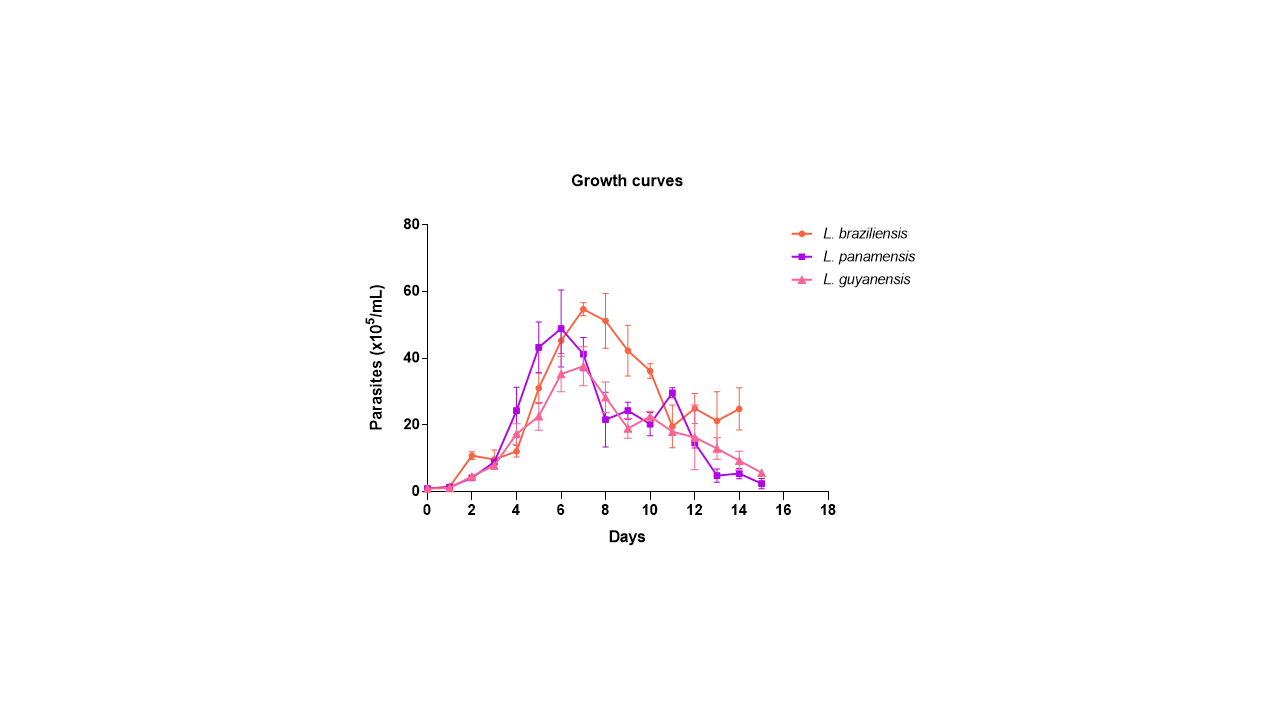

Supplement: S3 Fig — Every dot, square or triangle represents mean ± SD of biological triplicates for L. braziliensis, L. panamensis and L. guyanensis, respectively. Growth curves started by adding 1 x 10^5^ parasites/mL in 10 mL of Schnneider's medium supplemented with 10% of FBS. Parasites proliferation was evaluated every 24 h during two weeks by optical microscopy using a hemocytometer. (TIF) [file pntd.0008509.s003.tif]

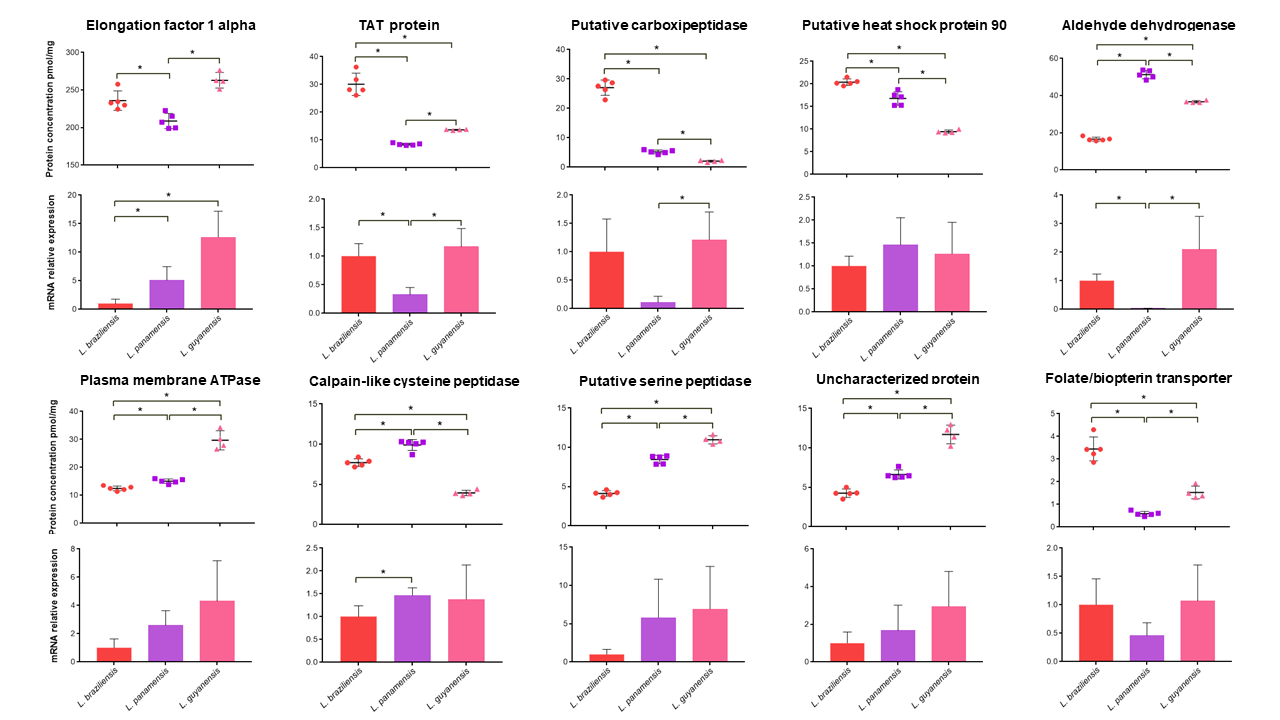

Supplement: S4 Fig — Values represent mean ± SD of protein concentration (pmol/mg) [n = 5 biological replicates for L. braziliensis (dots) and L. panamensis (squares) and n = 4 for L. guyanensis (triangles)]. * Statistical differences according to Perseus analysis (FDR < 0.01). Bars represent mean ± SD of mRNA relative levels determined by qPCR. * Significant differences by t test. (TIF) [file pntd.0008509.s004.tif]

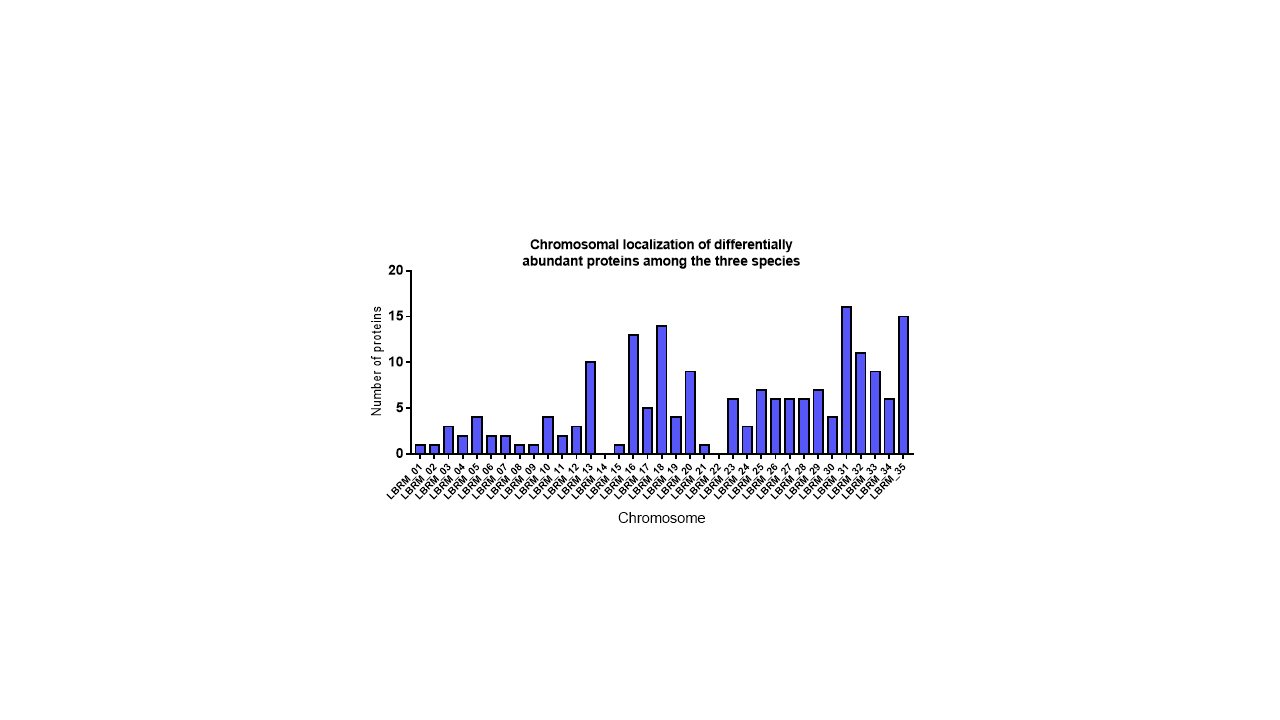

Supplement: S5 Fig — (TIF) [file pntd.0008509.s005.tif]

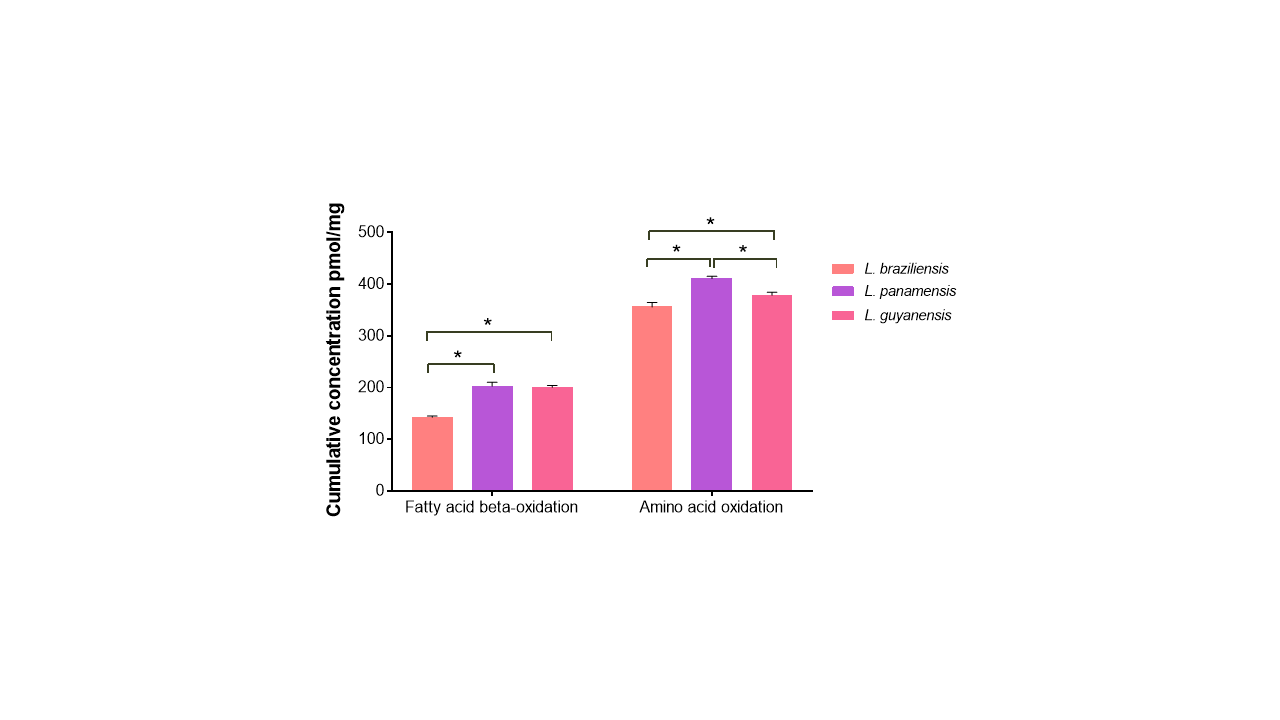

Supplement: S6 Fig — Bars show the mean of total sum of the concentration values in each species (n = 5 biological replicates for L. braziliensis and L. panamensis and n = 4 for L. guyanensis). Statistical differences by t test (p<0.01 Lb x Lb; p<0.005 Lb x Lg; p<0.0001 Lp x Lg). (TIF) [file pntd.0008509.s006.tif]

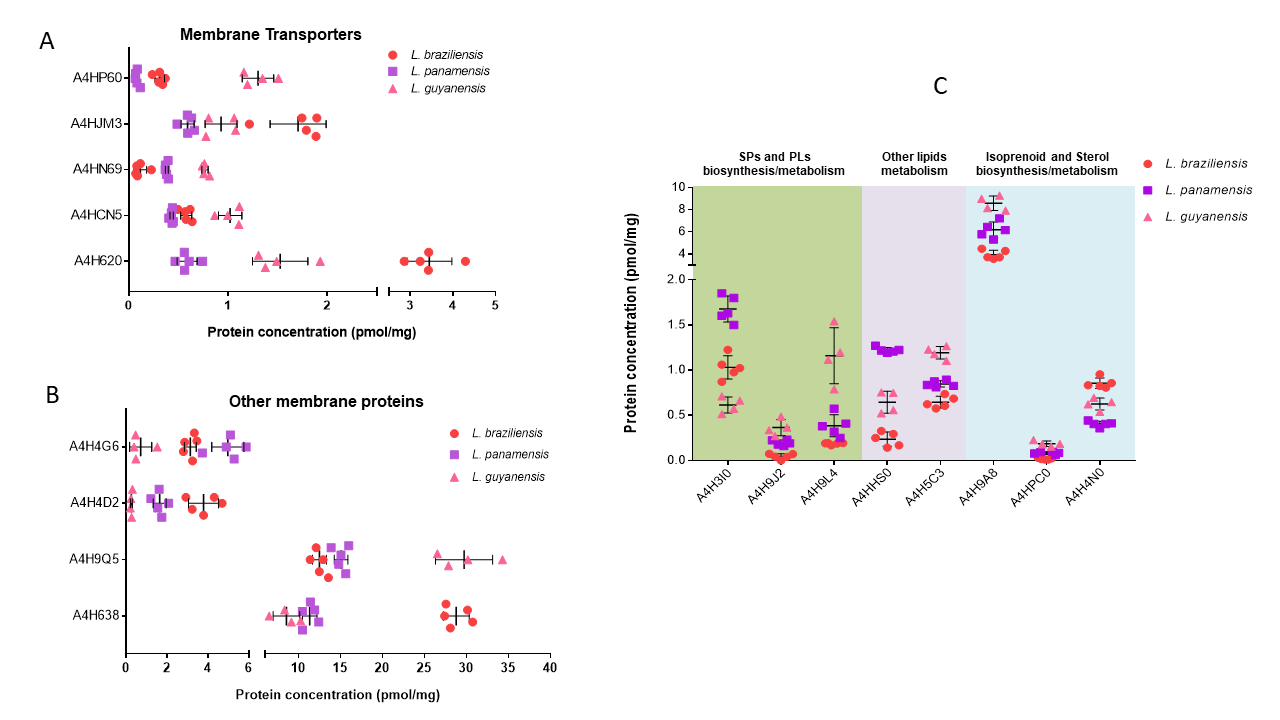

Supplement: S7 Fig — (A) Concentration values of different plasma membrane transporters. (B) Concentration values of different plasma membrane proteins. (C) Concentration values of proteins involved in biosynthesis/metabolism of sphingolipid (SL), phospholipid (PL) (green background), isoprenoid and sterols (blue background) and other lipids (purple background). Values represent mean of pmol/mg ± SD (n = 5 biological replicates for L. braziliensis and L. panamensis and n = 4 for L. guyanensis). All plotted values were statistically different across the three species in our proteomics dataset (FDR < 0.01) (S4 Table). (TIF) [file pntd.0008509.s007.tif]
