# Supplementary material for: In-depth quantitative proteomics uncovers specie-specific metabolic programs in Leishmania (Viannia) species
Source: PLoS Negl Trop Dis. 2020 Aug 17;14(8):e0008509. doi: 10.1371/journal.pntd.0008509 (PMC7451982; doi:10.1371/journal.pntd.0008509)
Supplement: Attachment — Submitted filename: Answer to Reviewers PNTD 2020.docx [file pntd.0008509.s012.docx]

|  | Ministério da Saúde  **FIOCRUZ - Fundação Oswaldo Cruz**  **Instituto Oswaldo Cruz**  **Laboratório de Pesquisa em Leishmaniose** |
| --- | --- |

## Rio de Janeiro, May 26, 2020

**Professors:**

Armando Jardim, PhD

Associate Editor

PLOS Neglected Tropical Diseases

Walderez Dutra

Deputy Editor

PLOS Neglected Tropical Diseases

**Dear Drs. Jardim and Dutra,**

Please find enclosed the answers, point by point, to the comments raised by the reviewers to the **Ms. No. PNTD-D-20-00350 “"In-depth quantitative proteomics uncovers specie-specific metabolic programs in *Leishmania* (*Viannia*) species"”**

In the revised version of the MS we have incorporated their suggestions as much as possible. We are grateful for the comments, these certainly contributed to improve our manuscript.

Sincerely yours,

**Patricia Cuervo, PhD**

Corresponding author

Laboratório de Pesquisa em Leishmaniose

Instituto Oswaldo Cruz, FIOCRUZ

Av. Brasil, 4365, Manguinhos. Pav. Leônidas Deane, sala 509.

CEP: 21040-360.

Rio de Janeiro, RJ.

Brazil.

*Av. Brasil 4365 – Manguinhos – Rio de Janeiro, RJ 21040-360 - Brazil*

*Tel.: (0055) (21)3865-8224 Fax: (0055) (21)3865-8195*

e-mail: [patricia.cuervo@fiocruz.br](mailto:patricia.cuervo@fiocruz.br)

Reviewer's Comments

Reviewer #1: This is a very nice paper. Its major contribution is the new and in-depth proteomic analysis of Leishmania (Viannia) species. Significantly, the data this study provides (about 7000 proteins per cell and their absolute abundance values) is significantly more comprehensive than in previous publications. This database justifies publication in PLoS NTD.

This is a very nice paper. Its major contribution is the new and in-depth proteomic analysis of Leishmania (Viannia) species. Significantly, the data this study provides (about 7000 proteins per cell and their absolute abundance values) is significantly more comprehensive than in previous publications. This database justifies publication in PLoS NTD.

The major weakness of this study is the biology behind the numbers. The authors sampled late log phase parasites (Materials and Methods). This is a border phase that require serious care to make sure that all the biological repeats were harvested at exactly same conditions.

For example, the change in the abundance in metabolic enzymes led the authors to conclude on the differences in the source of metabolic energy. In 1974, Mukkada et al. (Mukkada et al., 1974) and Opperdoes group 23 years later (Ter Kuile and Opperdoes, 1992) showed that log phase promastigotes utilize glucose as source of energy and when they reach stationary phase they move to proline and alanine, i.e. Kerb's cycle intermediates. It is likely that the observation made by the authors of this paper were due to timing of harvesting rather than real metabolic specificities.

The authors must provide a strong evodence that all three strains with their biological repeats were harvested at identical culture conditions. In mid log or stationary phases conditions are much more stable than late log (If I were to choose the experimental conditions, I would pick mid log phase). The authors should address this or avoid getting into conclusion on comparative metabolism.

We understand the reviewer's concern with this issue. To answer this question, we would like first to emphasize that the wide experience of our group with *Leishmania* culture, and particularly L. (Viannia), allows us to be sure that we have collected each biological replicate of each strain at the same point on the curve, i.e., in the late log phase. We know these parasites well; we have been working with these species for over 25 years and we have repeatedly made their growth curves in such a way that we are sure we have collected the parasites at the stage we stated. In addition, during our experiments, we decided to avoid any further manipulation of the parasites, such as purification with lectins or ficoll, in order to avoid unnecessary stresses that could generate changes in the abundance of proteins and, therefore, confounding our results.

Regarding differentiation markers that allow to distinguish between procyclic (log phase) and metacyclic (stationary phase) promastigotes, SHERP/HASPB, Meta1, tubulin beta, and GP63 have been described, all of them with greater expression in metacyclic than procyclic parasites, (Fong et al., 1984; Frommel et al., 1990; Bhaumik et al., 1991; Medina Acosta et al.,1993; Berberich et al, 1998; Uliana et al., 1999; Alce et al., 1999; McKean et al., 2001; Bellatin et al., 2002; Ramos et al., 2004; Jackson et al., 2006; Halle et al., 2009; Yao, 2009; Sádlová et al., 2010; Depledge et al, 2010; Ramos et al., 2011; Moore et al., 2011; Ouakad et al., 2011; Alcolea et al., 2016; Doehl et al., 2017; Giraud et al., 2019).

Among those markers, SHERP and HASPB are the best characterized to date and are the most used to differentiate between procyclic and metacyclic. It is important to clarify that HASPB and SHERP genes are encoded on the same segment of chromosome 23 in all Leishmania (Leishmania) species studied, but L. (Viannia) braziliensis present divergent sequences at the same location in the genome (Depledge et al., 2010). In L. braziliensis, the genes LbrM23_1110 and LbrM23_1120 code for SHERP/HASPB-like proteins (Depledge et al., 2010).

According to the reviewer, we harvested the parasites at different times of the differentiation process, *i.e*., L. braziliensis was collected in a log phase (mostly composed of procyclic promastigotes) and therefore it presented greater abundance of proteins related to glycolysis, whereas L. panamensis and L. guyanensis was collected at stationary phase (mostly composed of metacyclic parasites) and for this reason they have a greater abundance of proteins involved with amino acid metabolism and intermediates of the Krebs cycle. Following this reviewer's reasoning, the differentiation markers mentioned above should also have different abundances in the strains studied here. If this is so, the concentration of proteins such as SHERP/HASPB, Meta1, tubulin beta, and GP63 should be higher in L. panamensis and L. guyanensis, corroborating the reviewer's suspicion that they were collected in the stationary phase of the curve, differently of L. braziliensis.

As, unfortunately, in this pandemic moment, we have absolutely no conditions to carry out experiments at Fiocruz for checking the expression of these markers, **we will use our own proteomics dataset to answer the question of the reviewer**:

According to our dataset, SHERP/HASPB-like proteins were not detected in any of the species. Meta1 was also not detected in any species. The concentration of Tubulin beta was similar for the three species (255 pmol/mg, 241 pmol/mg, 245 pmol/mg) and the cumulative concentration of gp63, already reported in the manuscript, was higher for L. braziliensis than for the other two species. Based on this analysis, **we can affirm that none of the markers described for metacyclic promastigotes (of stationary phase) were increased in L. panamensis or L. guyanensis compared to L. braziliensis**. Based on this analysis, we can also state that the differences observed in the abundance of proteins involved in glycolysis or OxPhos in these species are not related to differences in the growth phase in which the parasites were collected.

In addition, as mentioned in the manuscript, the ribosomal subunits represented 12% of the total protein per cell, which is in agreement with the high activity of translation and synthesis of proteins observed in organisms in the logarithmic growth phase, i.e. dividing promastigotes. We did not observe differences among the percentage of total protein corresponding to ribosomal subunits among the species.

We included all those points in the revised version of the manuscript, please see lines 521-540:

“Further analysis of sugar uptake and oxygen consumption should be performed over the course of the parasites’ growth curve in order to obtain indisputable data on the metabolic traits of each *L.* (*Viannia*) species during parasite differentiation. Although parasites change their metabolic profile during differentiation from procyclic to metacyclic stages [66, 67], the differences between species observed here are not related with a discrepancy in the rate of differentiation of parasites because: (i) all replicates of each species were collected at the same point of the growth curve (late log phase, figure S3); (ii) proteins such as SHERP/HASPB and META1, used as differentiation markers because their abundance increases highly in metacyclic parasites (stationary phase) [68-71], were not detected in our dataset; (iii) proteins involved in translation and synthesis of proteins, which are processes very active in organisms in the log phase (*i.e*., dividing promastigotes) were highly abundant in our dataset; and (iv) differences in the protein abundance of ribosomal subunits, which are decreased in non-dividing metacyclic parasites (stat phase) [72], were not observed among the species studied here. Together, our data support the proposition that the three species of *L*. (*Viannia*) studied here have consistent differences in their energy metabolism. Our findings are in agreement with previous studies on the metabolome of different *L*. (*Leishmania*) species that demonstrated that *L. donovani*, *L. mexicana* and *L. major* are quite distinct in their metabolism [73]. Remarkably, large differences in consumption of amino acids were observed between those species [73]. Thus, potentially, the differences in energy metabolism sources could be a common trait in *Leishmania* spp.”

Based on those evidences, we would like to kindly ask the reviewer to reconsider his/her opinion about the results.

I have a few additional major and minor comments:

1. The title should be reconsidered.

Because following the suggestions of the reviewers we focused on the metabolic traits in the revised version of the manuscript, and also considering the reasons exposed above, we would like to ask this reviewer to reconsider the maintenance of the original title of our manuscript.

2. Abstract lines 32-33: "…Leishmania proteome remained mostly uncharacterized" and line 86 in Introduction. These statements are simply not true. Over 20 research papers have been published on Leishmania proteomics, each served its purpose and most of them used state-of-the-Art technologies. Together they are being used by the community. I assume that in 3 or 5 years someone else will use an advanced method that will undermine this "advanced" analysis. Modesty will be more appropriate here.

We apologize if our statement was uncomfortable; however, the statement made in the manuscript is not the product of a lack of modesty, that statement is a fact. The full sentence is: (sic) “*During the last decade, there have been impressive efforts for sequencing the genome of most of the pathogenic Leishmania spp. as well as hundreds of strains, but large-scale proteomics analyses did not follow these achievements and the Leishmania proteome remained mostly uncharacterized.”* This statement is a fact.

In the manuscript we summarize the contributions from studies that, so far, made the most complete descriptions of the *Leishmania* proteome. None of them described more than 40-50% of the proteome and none of these works did a thorough quantitative analysis. These are facts. We did not claim that these works did not contribute important information. What we clearly stated is that, compared to the Leishmania genome, which has been studied for more than 15 species and thousands of strains, the proteome has only been partially described for few species and no more than for a couple of dozens of strains. Quantitative proteomic data are available only for *L. mexicana* and *L. donovani* and these data cover 20-30% of the proteome. With our statement we want to raise the awareness of the scientific community about the need to study the *Leishmania* proteome with the same enthusiasm as the genome.

3. Introduction line 50 "…species-specific differentiation…" did you mean "differences?

It was corrected.

4. Methods lines 131-132: see the paragraph above. Indicate how did you make sure parasites are all at exactly same growth phase conditions.

Please, see answer above.

5. The authors have not provided the complete peptide list for each of the three Leishmania (Viannia) species. This is one of the first shotgun proteomic studies I have come across in many years where the authors have not provided peptide information as a part of the manuscript.

Because of the size, it is rather uncommon to provide a peptide list as a part of the manuscript. The peptides are accessible to the reviewer (and later to community) at PRIDE. See details exclusive for reviewers:

Reviewer account details:

Username: reviewer33371@ebi.ac.uk

Password: SpNW5rkl

Project Name: Comprehensive quantitative analysis of the proteome of Leishmania (Viannia) species

Project accession: PXD017696

6. If one looks at supplementary table 2 it is not possible to identify a single L. panamensis or L. guyanensis accession from UniProt database. However, in the methods section proteomic data analysis the authors state that "The data were analyzed with MaxQuant Software using the L. braziliensis, L. guyanensis and L. panamensis protein sequences available at UniProt database." It is not very clear what database dependent searches have been undertaken by the authors and this bring to question the veracity of study and identifications. The authors should make clear if they have searched the MS data from all the three Leishmania species only against a L. braziliensis database? Or they have searched a combined uniprot database of the three species, in that case why only L. braziliensis UniProt Ids are shown in the supplementary data and not the other two species.

We used only L. braziliensis FASTA sequences. We apologize for this mistake. The information was corrected in the revised version of the manuscript. Please see corrected information, line 161:

“The data were analyzed with MaxQuant Software using the *L. braziliensis* protein sequences available at UniProt database.”

7. Continuing with the above question have the authors looked at species specific genes (which would be handful) identified based on genome sequencing data which are truly unique to each L. braziliensis, L. guyanensis and L. panamensis species used in the study. These genes would make each of species different from one another. This shouldn’t be mistaken for the proteomic expression analysis that the authors undertook where they have identified 25 (L. braziliensis), 2 (L. guyanensis) and 3 (L. panamensis) species specific protein expression as shown in Figure 3A.

As I mentioned before, we used only L. braziliensis FASTA sequences for protein identification. We agree with this reviewer that species-specific genes should not be mistaken with species-specific proteins detected in any specific condition. For this reason, we did not discuss anything related to these 25 (L. braziliensis), 2 (L. guyanensis) or 3 (L. panamensis) proteins, nor did we affirm that these proteins are species-specific "markers". We only showed the result because it was a fact; it was what we observed in the conditions used here.

8. In the results section: How do the authors differentiate between close paralog which are more than 95% identical and peptide assignments. The authors seem to be overestimating their protein identifications. If they are suggesting that each paralog in a multi-gene family are translated equally considering Leishmania gene expression is regulated post-transcriptionally. They should clarify this aspect of in case of multi-gene families or genes with more than one paralog.

The software used, MaxQuant, identifies protein groups according to peptide matches, i.e. according to developers, if all detected peptides of protein A can also belong to protein B, A and B form one protein group, for this reason proteins that have only shared peptides (i.e., proteins that cannot be unambiguously identified by unique peptides) are grouped in one protein group. In addition, the software analyzes “unique peptide” matches, that is peptides that are an exclusive signature of each protein. If those unique peptides are successfully assigned to one single protein, this single protein will be alone in a protein group. For these reasons, our table S2 is named Protein groups identification and it presents specific columns for peptides, razor + unique peptides and unique peptides. Razor peptides are peptides which are shared between protein groups (e.g. protein X and protein Y are unambiguously identified via unique peptides, but nevertheless share a common peptide). There is no software for protein identification that considers gene regulation.

9. The authors should provide a complete list of genes including gene ids which have one or more paralogs in each of the Leishmania species they have used in their analysis and the peptides identified in the present study that map to or are shared across the different paralogs in each Leishmania species.

Please find attached the table requested. We included the proteins encode by paralog genes according to the list of paralog genes retrieved from TriTryp (L. braziliensis and L. panamensis) and Coughlan et al., 2018 (L. guyanensis). This table shows that every protein (protein group) in each paralog group has at least one unique peptide and/or razor peptide used for identification.

10. On line 269 the authors state “our results show that global protein abundances obtained by deep proteomics are able to clearly distinguish the three species, in a similar way that deep genome sequencing allows such distinction [9,12].” What are the species specific proteins have been identified by the authors, which are unique to just one species but not identified. The authors should describe one section in the results and discussion regarding these species-specific proteins identified in the study. These can be the ones that make one species of Leishmania have the specific pathogenicity not found in the other species. Considering more than 98% of genes are conserved across the old and new world species this should not be a difficult task for the authors.

As the reviewer highlighted, we stated that global protein abundances **obtained by deep quantitative proteomics** are able to clearly distinguish the three species and we would like to reinforce that it is the set of several hundred proteins that defines this clear separation and not just a small number of proteins. As we mentioned before, we are very careful in order to do not attribute to a specific group of proteins "the responsibility of a phenotype", since we are convinced that this would be a very reductionist view. This is especially important when it comes to proteins, given the vast amount of modifications that these molecules can undergo and taking into account that the genomic sequencing cannot foresee such modifications.

Among the reasons for not making such assumptions, we also consider that the fact of not identifying a protein by mass spectrometry does not mean that the protein was not in the sample in the condition we studied here. Nor can we assume that the "species specific" proteins identified here define pathogenicity since phenotypes are defined by a complex group of factors that must include different proteins, modifications of these, in addition to other molecules. For these reasons we choose not to discuss or highlight these proteins. We hope the reviewer understands our position and reconsiders his/her request.

11. In Figure 5 the authors should provide density plots for metallopeptidase activity bands in the zymographic analysis for the three species.

Please see the densitometry below. However, following the suggestions of reviewers 1 and 3, regarding shortening the manuscript, the main findings in figures 5 to 7 were condensed in a new supplementary figure (S7 Fig). Their respective texts were removed from the manuscript leaving only a brief paragraph that summarize the results in S7 Fig.

The zymography, GP63, transporters, sterols, SLs and PLs cumulative concentration plots were removed from the revised version.

12. For the manuscript whose sole claim to glory is the depth of the protein identifications. The authors have provided very poor images which appear grainy at the best and pixelated at worst. The authors should re-export higher dpi images from MaxQuant or Perseus software and provide it again.

Regarding the images, we improved the PCA from Perseus. All the figures were submitted to the PNTD - PACE digital diagnostic tool, and all passed the quality test.

We reiterate that we do not claim glory with this work. This is a honest and serious study that we hope can contribute new information on proteins that could potentially be exploited for new diagnostic strategies. Our hope is that groups with more resources than us can explore this data and use it to improve diagnosis in the Americas, which to date, continues to be a problem.

13. The authors have submitted their data to Proteomexchange repository. However, the data is not made publicly available. Even as a reviewer I am not able to access the data. The authors should make their data in Proteomexchange accessible to public or should provide access to the reviewers. Hopefully they have provided processed searched data files and also raw data files as part submission to Proteome exchange if not they should do it.

The raw files and peptide list are accessible to the reviewer (and later to community) at PRIDE. See details exclusive for reviewers:

Reviewer account details:

Username: reviewer33371@ebi.ac.uk

Password: SpNW5rkl

Project Name: Comprehensive quantitative analysis of the proteome of Leishmania (Viannia) species

Project accession: PXD017696

All other processed data are available within this manuscript in the main tables and in the supplementary tables 2 and 4.

14. The results and discussion section of the manuscript is very lengthy, exhaustive and at times it quite mind-numbing to read such a lengthy manuscript. The authors should shorten the results and discussion section of the manuscript and only describe the most important finding in a succinct manner.

Thanks for the comment. Following the suggestion of the reviewers, the results and discussion section was shortened, focusing on the metabolic finding; the main findings in figures 5 to 7 were condensed in one supplementary figure (new S7 figure) and the corresponding texts were removed from the manuscript leaving only a brief paragraph that summarize the results in S7 Fig. Please see new S7 figure and the modified version of the manuscript, lines 552-561:

“In addition to the differences in metabolism, we observed that concentration values of many proteins annotated as integral components of membrane, including transporters, surface antigens-like proteins, plasma membrane ATPases, and GP63, as well as proteins related to the metabolism of plasma membrane’ lipids were significantly different across the three *L.* (*Viannia*) species (S4 Table, S7 Fig) and allowed distinguishing the three species studied here. Together, our results reveal a species-specific pattern of protein abundances that might be contributing to specific host adaptation and differentiation. Such remarkable differences could be exploited for better and suitable diagnosis tools. The mass spectrometry proteomics data have been deposited to the ProteomeXchange Consortium via the PRIDE [96] partner repository with the dataset identifier PXD017696.”

15. Figure legends and tables are smack in the middle of the manuscript which disturbs the flow. The authors should know that at later time point the manuscript once accepted is typeset. So, the figures and tables could be having been provided at the end of the manuscript

The legends of the figures and tables in the middle of the manuscript are in accordance with the submission guidelines of the journal, at the manuscript organization section, please see: <https://journals.plos.org/plosntds/s/submission-guidelines#loc-manuscript-organization>.

Reviewer #2: All data are clearly presented in figures and tables, and properly described in the text.

Thanks.

Reviewer #3: see comment 2 and 3 in editorial and data representation modifications section.

Thanks.

--------------------

Conclusions

-Are the conclusions supported by the data presented?

-Are the limitations of analysis clearly described?

-Do the authors discuss how these data can be helpful to advance our understanding of the topic under study?

-Is public health relevance addressed?

Reviewer #1: See above

Please see our answers above.

Reviewer #2: As detailed below, the study has two main flaws that challenge the major findings on (i) comparative analysis between parasite species based on absolute quantification and (ii) the identification of species-specific differences in energy metabolism.

Please see our answers below.

Reviewer #3: see general comments.

--------------------

Editorial and Data Presentation Modifications?

Use this section for editorial suggestions as well as relatively minor modifications of existing data that would enhance clarity. If the only modifications needed are minor and/or editorial, you may wish to recommend “Minor Revision” or “Accept”.

Reviewer #1: Peptide list should be free to the community. See coment 5

Please see our answers above.

Reviewer #2: (No Response)

Reviewer #3: 1. Differences in abundance of metabolic enzymes in the parasites is clearly dependent on differentiation. In the chosen set up this means, it is critically dependent on the growth characteristics of the chosen strains. Thus, I would expect authors to analyse, or at least stress that for conclusive data, the sugar uptake and oxygen consumption measurements should be performed over the course of a full growth curve in vitro for all the isolates.

Thanks for the comment. Unfortunately, in this pandemic moment, we have absolutely no conditions to carry out experiments at Fiocruz for measuring sugar uptake and oxygen consumption along the parasite’s growth curve. We have modified the text to include the suggestion of this reviewer about this issue. Please see lines 521-540:

“Further analysis of sugar uptake and oxygen consumption should be performed over the course of the parasites’ growth curve in order to obtain indisputable data on the metabolic traits of each *L.* (*Viannia*) species during parasite differentiation. Although parasites change their metabolic profile during differentiation from procyclic to metacyclic stages [66, 67], the differences between species observed here are not related with a discrepancy in the rate of differentiation of parasites because: (i) all replicates of each species were collected at the same point of the growth curve (late log phase, figure S3); (ii) proteins such as SHERP/HASPB and META1, used as differentiation markers because their abundance increases highly in metacyclic parasites (stationary phase) [68-71], were not detected in our dataset; (iii) proteins involved in translation and synthesis of proteins, which are processes very active in organisms in the log phase (*i.e*., dividing promastigotes) were highly abundant in our dataset; and (iv) differences in the protein abundance of ribosomal subunits, which are decreased in non-dividing metacyclic parasites (stat phase) [72], were not observed among the species studied here. Together, our data support the proposition that the three species of *L*. (*Viannia*) studied here have consistent differences in their energy metabolism. Our findings are in agreement with previous studies on the metabolome of different *L*. (*Leishmania*) species that demonstrated that *L. donovani*, *L. mexicana* and *L. major* are quite distinct in their metabolism [73]. Remarkably, large differences in consumption of amino acids were observed between those species [73]. Thus, potentially, the differences in energy metabolism sources could be a common trait in *Leishmania* spp.”

2. GP63 is notoriously showing lower than expected abundance in proteomic data sets of the parasites and this may relate to solubilisation problems since also in their approach lysates are cleared before processing them further for eventual peptide determination by MS. Such a technical explanation for inconsistencies is neither considered nor discussed. As is the general problem whether e.g. membrane proteins are indeed as quantitatively sampled as soluble proteins or become systematically under-represented in such data sets. This should be discussed.

It is true that membrane proteins are underrepresented in proteomic studies because the methods used for sample preparation are not efficient in extraction and digestion of this kind of proteins. However, the introduction of SDS, followed by urea washes and multienzyme digestion, in the FASP method for sample preparation clearly improves the sensitivity for membrane proteins in proteomics studies (Wiśniewski et al., 2009, doi:10.1021/pr900748n).

It is known that 20-30% of eukaryotic genes code for membrane proteins (Wallin and von Heijne, 1998 doi:10.1002/pro.5560070420). That percentage corresponds to approximately 2000 proteins in the Leishmania genome (~8000 proteins). Here, we identified 1096 membrane proteins which corresponds to 16% proteins identified in our dataset and about 50% of total expected membrane proteins. Taking into account that 40-50% of the *Leishmania* genome has no functional annotation and that ~70% does not have annotation of cellular component, it is indeed probably that the representation of membrane proteins in our dataset is much higher, but we don’t know because of the lack of annotations. Based in our results, we do not believe that membrane proteins are underrepresented in our dataset. We overcame solubilization problems during sample preparation because we used SDS 4%, which is the most potent solubilizing agent known, and we combined this procedure with multienzyme digestion. Together, these procedures improved the sensitivity of our study.

Regarding the abundance of GP63, although it is not found among the top 20 or top 100 most abundant protein groups in terms of copy number (or protein concentration), it is in the 204 rank, with ~20000 – 67000 copy numbers, 8 – 28 pmol/mg and intensities between 3.0-6.0E+10 (please see Table S2). The intensity value is similar to that found for GP63 in other gel-free and label-free quantitative proteomics studies (Tupperwar et al., 2019 10.1128/mSphere.00625-19), reinforcing that differences observed are not related to underrepresentation of membrane proteins.

3. Data and text regarding figures 5-7 could be either moved to the supplementary section or left out altogether, since they simply illustrate ideas on how to develop hypothesis that try to mine and exploit the really great proteome dataset. I would suggest leaving this exercise to the interested reader and scientific community to which this resource is becoming available. Also, this will leave time to substantiate the authors’ own hypothesis without presenting examples that are not yet revealing.

Thanks for the comment. Following the suggestion of the reviewers, the results and discussion section was shortened, focusing on the metabolic finding; the main findings in figures 5 to 7 were condensed in one supplementary figure (new S7 figure) and the corresponding texts were removed from the manuscript leaving only a brief paragraph that summarize the results in S7 Fig. Please see new S7 figure and the modified version of the manuscript, lines 552-561:

“In addition to the differences in metabolism, we observed that concentration values of many proteins annotated as integral components of membrane, including transporters, surface antigens-like proteins, plasma membrane ATPases, and GP63, as well as proteins related to the metabolism of plasma membrane’ lipids were significantly different across the three *L.* (*Viannia*) species (S4 Table, S7 Fig) and allowed distinguishing the three species studied here. Together, our results reveal a species-specific pattern of protein abundances that might be contributing to specific host adaptation and differentiation. Such remarkable differences could be exploited for better and suitable diagnosis tools. The mass spectrometry proteomics data have been deposited to the ProteomeXchange Consortium via the PRIDE [96] partner repository with the dataset identifier PXD017696.”

4. A major aspect of the rules governing protein abundance in these parasites has been translational bias in codon usage (e.g. see Jeacock et al. Elife. 2018 Mar 15;7. pii: e32496. doi: 10.7554/eLife.32496). It seems that the presented datasets lend themselves perfectly to analyse this and, possibly, this can qualify also the answer to comment 2 above.

Thanks for the comment. Indeed, this is a very interesting point and we agree that our dataset present a good opportunity for analyzing the codon usage bias in these species and how it regulates the protein abundance observed here. We believe that the study of this subject may certainly be conducted in the future.

--------------------

Summary and General Comments

Use this section to provide overall comments, discuss strengths/weaknesses of the study, novelty, significance, general execution and scholarship. You may also include additional comments for the author, including concerns about dual publication, research ethics, or publication ethics. If requesting major revision, please articulate the new experiments that are needed.

Reviewer #1: The manuscript by Patricia Cuervo’s group utilizes a label free quantitative proteomic approach to map the ultra-deep and to compare the proteomes of Leishmania (Viannia) species namely L. braziliensis, L. panamensis and L. guyanensis. The authors mapped more than 80% of parasite proteins using a multi-enzyme digestion filter aided sample preparation (FASP) method and coupled with analysis using a high accuracy Orbitrap Q-Exactive HF mass spectrometer. The authors have successfully applied previously established methods such as total protein approach (TPA) to quantitate abundance of proteins based on spectral intensities and histone ruler approach to estimate protein copy number per Leishmania cell. As indicated in the title, the authors report on differences in energy metabolism precursors between the three species. While L. braziliensis rely on glycolysis, the other two utilize mitochondrial metabolites as source for metabolic energy.

This is a very nice paper. Its major contribution is the new and in-depth proteomic analysis of Leishmania (Viannia) species. Significantly, the data this study provides (about 7000 proteins per cell and their absolute abundance values) is significantly more comprehensive than in previous publications. This database justifies publication in PLoS NTD.

We answered all these questions already. Please see our answers above.

The major weakness of this study is the biology behind the numbers. The authors sampled late log phase parasites (Materials and Methods). This is a border phase that require serious care to make sure that all the biological repeats were harvested at exactly same conditions. For example, the change in the abundance in metabolic enzymes led the authors to conclude on the differences in the source of metabolic energy. In 1974, Mukkada et al. (Mukkada et al., 1974) and Opperdoes group 23 years later (Ter Kuile and Opperdoes, 1992) showed that log phase promastigotes utilize glucose as source of energy and when they reach stationary phase they move to proline and alanine, i.e. Kerb's cycle intermediates. It is likely that the observation made by the authors of this paper were due to timing of harvesting rather than real metabolic specificities. The authors must provide a strong evodence that all three strains with their biological repeats were harvested at identical culture conditions. In mid log or stationary phases conditions are much more stable than late log (If I were to choose the experimental conditions, I would pick mid log phase). The authors should address this or avoid getting into conclusion on comparative metabolism.

Please see our answer above.

I have a few additional major and minor comments:

1. The title should be reconsidered. Please see our answers above.

2. Abstract lines 32-33: "…Leishmania proteome remained mostly uncharacterized" and line 86 in Introduction. These statements are simply not true. Over 20 research papers have been published on Leishmania proteomics, each served its purpose and most of them used state-of-the-Art technologies. Together they are being used by the community. I assume that in 3 or 5 years someone else will use an advanced method that will undermine this "advanced" analysis. Modesty will be more appropriate here. Please see our answers above.

3. Introduction line 50 "…species-specific differentiation…" did you mean "differences? Please see our answers above.

4. Methods lines 131-132: see the paragraph above. Indicate how did you make sure parasites are all at exactly same growth phase conditions. Please see our answers above.

5. The authors have not provided the complete peptide list for each of the three Leishmania (Viannia) species. This is one of the first shotgun proteomic studies I have come across in many years where the authors have not provided peptide information as a part of the manuscript. Please see our answers above.

6. If one looks at supplementary table 2 it is not possible to identify a single L. panamensis or L. guyanensis accession from UniProt database. However, in the methods section proteomic data analysis the authors state that "The data were analyzed with MaxQuant Software using the L. braziliensis, L. guyanensis and L. panamensis protein sequences available at UniProt database." It is not very clear what database dependent searches have been undertaken by the authors and this bring to question the veracity of study and identifications. The authors should make clear if they have searched the MS data from all the three Leishmania species only against a L. braziliensis database? Or they have searched a combined uniprot database of the three species, in that case why only L. braziliensis UniProt Ids are shown in the supplementary data and not the other two species. Please see our answers above.

7. Continuing with the above question have the authors looked at species specific genes (which would be handful) identified based on genome sequencing data which are truly unique to each L. braziliensis, L. guyanensis and L. panamensis species used in the study. These genes would make each of species different from one another. This shouldn’t be mistaken for the proteomic expression analysis that the authors undertook where they have identified 25 (L. braziliensis), 2 (L. guyanensis) and 3 (L. panamensis) species specific protein expression as shown in Figure 3A. Please see our answers above.

8. In the results section: How do the authors differentiate between close paralog which are more than 95% identical and peptide assignments. The authors seem to be overestimating their protein identifications. If they are suggesting that each paralog in a multi-gene family are translated equally considering Leishmania gene expression is regulated post-transcriptionally. They should clarify this aspect of in case of multi-gene families or genes with more than one paralog. Please see our answers above.

9. The authors should provide a complete list of genes including gene ids which have one or more paralogs in each of the Leishmania species they have used in their analysis and the peptides identified in the present study that map to or are shared across the different paralogs in each Leishmania species. Please see our answers above.

10. On line 269 the authors state “our results show that global protein abundances obtained by deep proteomics are able to clearly distinguish the three species, in a similar way that deep genome sequencing allows such distinction [9,12].” What are the species specific proteins have been identified by the authors, which are unique to just one species but not identified. The authors should describe one section in the results and discussion regarding these species-specific proteins identified in the study. These can be the ones that make one species of Leishmania have the specific pathogenicity not found in the other species. Considering more than 98% of genes are conserved across the old and new world species this should not be a difficult task for the authors. Please see our answers above.

11. In Figure 5 the authors should provide density plots for metallopeptidase activity bands in the zymographic analysis for the three species. Please see our answers above.

12. For the manuscript whose sole claim to glory is the depth of the protein identifications. The authors have provided very poor images which appear grainy at the best and pixelated at worst. The authors should re-export higher dpi images from MaxQuant or Perseus software and provide it again. Please see our answers above.

13. The authors have submitted their data to Proteomexchange repository. However, the data is not made publicly available. Even as a reviewer I am not able to access the data. The authors should make their data in Proteomexchange accessible to public or should provide access to the reviewers. Hopefully they have provided processed searched data files and also raw data files as part submission to Proteome exchange if not they should do it. Please see our answers above.

14. The results and discussion section of the manuscript is very lengthy, exhaustive and at times it quite mind-numbing to read such a lengthy manuscript. The authors should shorten the results and discussion section of the manuscript and only describe the most important finding in a succinct manner. Please see our answers above.

15. Figure legends and tables are smack in the middle of the manuscript which disturbs the flow. The authors should know that at later time point the manuscript once accepted is typeset. So, the figures and tables could be having been provided at the end of the manuscript

Please see our answers above.

Reviewer #2: The manuscript "In-depth quantitative proteomics uncovers specie-specific metabolic programs in Leishmania (Viannia) species" by Pinho et al. provides quantitative insight into the proteomes of three Leishmania strains, one each corresponding to L. braziliensis, L. panamensis and L. guyanensis species. The authors compare the results both by relative quantification on total protein levels, as well as by absolute quantification using histone expression as a biological ruler to calculate protein concentration and copy number on a cellular level. Based on these results, the authors investigate energy metabolism in these parasites and provide biochemical evidence that L. braziliensis energy production relies on glycolysis, while L. panamensis and L. guyanensis seem to depend mainly on mitochondrial respiration. The manuscript is well written and provides deep proteomic insight into three Leishmania species of medical importance. As such it is a new and very useful resource for the larger parasitology community. However, the study has two main flaws that challenges the major findings:

Thanks for the comment. Before to answer the next two questions, we would like to clarify that **we do not made relative quantifications**. Both methods used in our analysis, “total protein approach (TPA)” and “histone ruler”, are methods for absolute quantification (Methods Enzymol 2017;585:49-60. doi: 10.1016/bs.mie.2016.10.002). The “total protein approach” allows the quantification of absolute values of concentration based on the total intensity of mass spectra signals (whole dataset). Thus, the calculations of protein concentrations are not based on histones.

The “histone ruler” method allows the calculation of absolute protein copy numbers based on the protein abundance specific for histones calculated by TPA. The “histone ruler” is exclusively used for copy number calculations.

First, the absolute quantification is based on calculations assuming that the three parasite strains are disomic for all chromosomes and that histones are expressed equally across these strains. The frequent aneuploidies observed in parasites in culture and the triploid state of L. braziliensis (trisomic state of all chromosomes, with higher ploidies observed in few chromosomes, see Rogers et al, PMID: 22038252) calls these assumptions into question. Experimental proof of the copy number and expression levels for the histone(s) used for normalization is required, for example by HTseq or RTqPCR analysis to assess gene copy number, and Western Blot analysis of histone expression levels (various antibodies are available in the community).

As explained above, the “histone ruler” was used for calculations of protein copy number. We indeed used a parsimonious 2n assumption for calculation of copy numbers because, as we explained in the manuscript, it has been shown that the chromosome number in *Leishmania* sp. (i) varies among species and strains [7,8,12,13]; (ii) varies in response to external stimuli [13,15,16, 41,42]; (iii) is present as a mosaic, i.e., different chromosomes have different ploidy [43]; (iv) is highly variable in vitro-cultivated strains but does not seem to vary as much in intracellular parasites from mammal hosts [17,42], and (v) has been barely studied in species of the subgenus *L.* (*Viannia*). All the references, which include the one of Rogers et al., were cited in the original manuscript.

Also, as we stated in the manuscript, we are aware that these calculations could vary according to the ploidy of the genome but **the ploidy status of the strains here studied is unknown**. Thus, if the DNA content in cells or parasites deviate from 2n, and it is known, the actual copy numbers can be simply recalculated using this value. This is the power of the method.

Our **calculations for copy number are not assuming that histones are expressed equally across** the strains. The calculations taken into account the mass spectra intensity values of histones individually for each replicate of each strain. Please **see S2 table, histone ruler sheet.** So, the value reflects, on the contrary to the reviewer understanding, the very specific expression in each strain.

Unfortunately, in this pandemic moment, we have absolutely no conditions to carry out experiments of HTseq or more RTqPCR, or Western blots as suggested. In addition, both HTseq or RTqPCR do not provide information on protein abundance and it has been extensively demonstrated that mRNA levels and protein abundance have poor correlation in *Leishmania* as well as in other eukaryotes (Mol Biochem Parasitol. 2007; 152(1):35-46; FASEB J. 2011;25(2):515-25; Science. 2010;329(5991):533-8; Nat Rev Genet. 2012;13(4):227-32; Cell 2016;165(3):535-50; Stem Cell Reports 2020;14(5):972-988). We indeed presented in the S4 Fig., the poor relationship between transcript levels and protein abundance for 10 different genes using RTqPCR. Additionally, Western blots are only occasionally quantitative but are less sensitive than mass spectrometry.

**It is of the most important to note that all of our results and discussion of metabolic traits are based on protein concentration values, which are much more appropriate than copy numbers when analyzing biological activities.** As explained before, concentration values are calculated based on the total intensity of mass spectra signals, not on the histones.

Second, even though the differences in energy metabolism between strains is potentially very interesting, the analysis of just a single strain per parasite species raises the question whether this observation is just a strain- rather than species-specific phenomenon. Indeed, genomic and phenotypic variability between isolates of a given species is one of the hallmarks of Leishmania. Thus, species-specific differences need to be validated by analysing at least three different strains (best of distinct geographic origin) per parasite species using the established biochemical approaches.

We agree with this reviewer that more strains should be tested. In fact, we mention it in various parts of the manuscript. We look forward to doing so in the near future. Our hypothesis when we started this study was that in-depth quantitative proteomic analysis is capable of distinguishing *Leishmania* species and, in fact, we fairly demonstrated this. In making such a demonstration, among the relevant differences that we described, we observed that energy metabolism may be different between these species. If this is a species-specific characteristic, it is now a new hypothesis that we certainly want to demonstrate (or deny) in the future. However, again, we consider that the question about the **ability of deep quantitative proteomics to distinguish between *Leishmania* species**, which motivated our study, was answered fairly and consistently in this manuscript.

We hope this reviewer reconsiders his/her opinion as in fact we developed a technically sound study around the original question we asked.

Reviewer #3: Pinho and colleagues report on their proteomic analysis of New World Leishmania species of the Viannia complex. The authors are commended for this work, in particular, for significantly expanding the proteomic database in this field.

The strengths of the study are the application of a state of the art, label free quantitative approache to analyse the proteome of in vitro grown parasite isolates of L. braziliensis, L. guayanensis and L. panamensis. This is a significant, stand alone contribution to our knowledge base and contributes information on nearly 80% of the genomic information derived, predicted protein ensemble that makes up these parasites.

Weaknesses are mostly related to the mentioned issues and, in comparison to the strength, are considered minor.

Thanks.

--------------------

PLOS authors have the option to publish the peer review history of their article (what does this mean?). If published, this will include your full peer review and any attached files.

If you choose “no”, your identity will remain anonymous but your review may still be made public.

Do you want your identity to be public for this peer review? For information about this choice, including consent withdrawal, please see our Privacy Policy.

Reviewer #1: No

Reviewer #2: No

Reviewer #3: No

Figure Files:

While revising your submission, please upload your figure files to the Preflight Analysis and Conversion Engine (PACE) digital diagnostic tool, https://pacev2.apexcovantage.com. PACE helps ensure that figures meet PLOS requirements. To use PACE, you must first register as a user. Then, login and navigate to the UPLOAD tab, where you will find detailed instructions on how to use the tool. If you encounter any issues or have any questions when using PACE, please email us at figures@plos.org.

It was done and all figures pass the quality test.

Data Requirements:

Please note that, as a condition of publication, PLOS' data policy requires that you make available all data used to draw the conclusions outlined in your manuscript. Data must be deposited in an appropriate repository, included within the body of the manuscript, or uploaded as supporting information. This includes all numerical values that were used to generate graphs, histograms etc.. For an example see here: <http://www.plosbiology.org/article/info%3Adoi%2F10.1371%2Fjournal.pbio.1001908#s5>.

Data was deposited in the PRIDE repository.

Reproducibility:

To enhance the reproducibility of your results, PLOS recommends that you deposit laboratory protocols in protocols.io, where a protocol can be assigned its own identifier (DOI) such that it can be cited independently in the future. For instructions see https://journals.plos.org/plosntds/s/submission-guidelines#loc-methods
